# Supplementary material for: Comparative analysis based on shared amplicon sequence variants reveals that cohabitation influences gut microbiota sharing between humans and dogs
Source: Front Vet Sci. 2024 Oct 7;11:1417461. doi: 10.3389/fvets.2024.1417461 (PMC11491291; doi:10.3389/fvets.2024.1417461)
Supplement: Supplementary file 1 [file Data_Sheet_1.DOCX]

***Supplementary Material***

1. **Supplementary Figures**


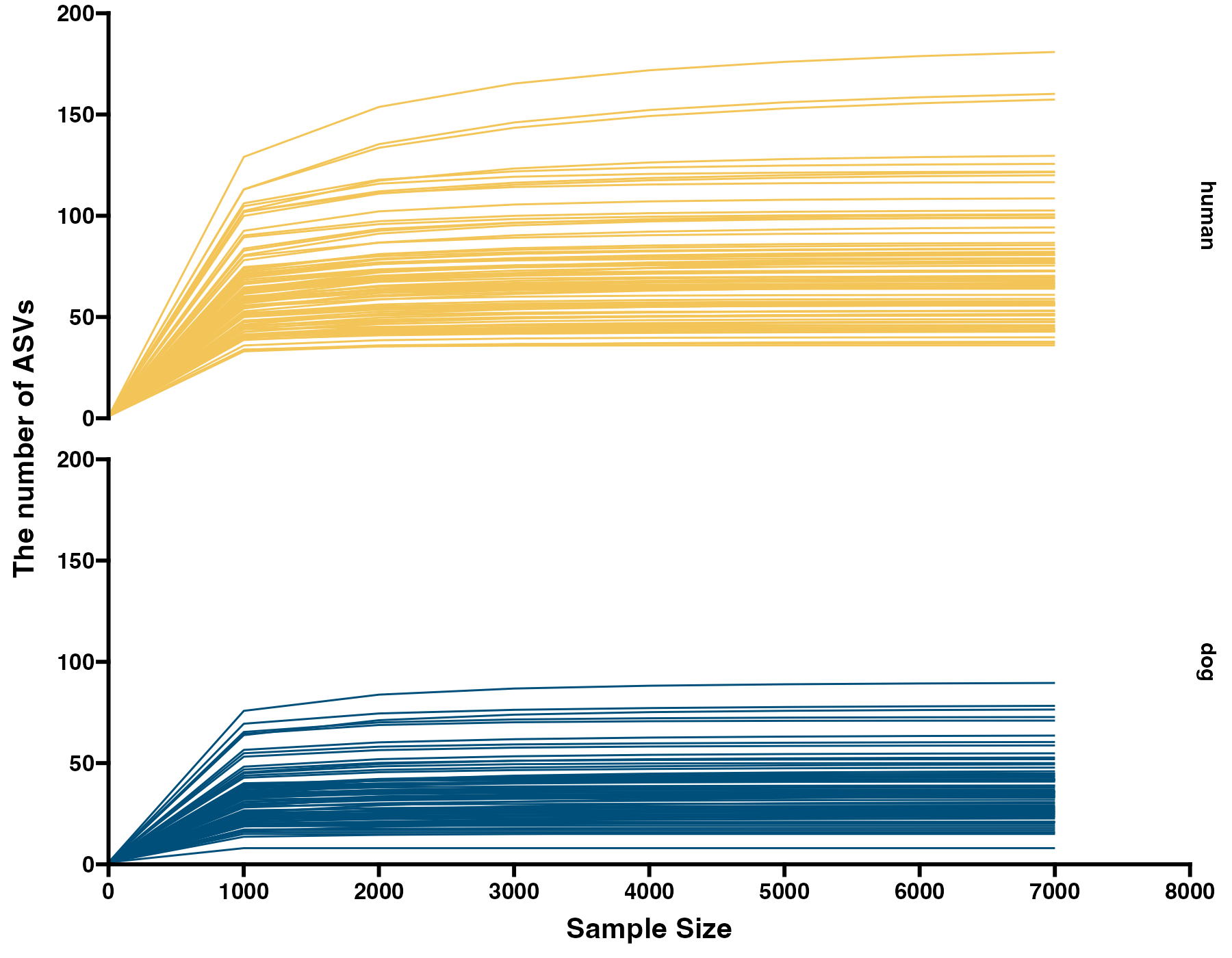


Supplementary Figure 1. Rarefaction curves for the number of observed ASVs by sample size.

Rarefaction curves for the human and dog fecal samples. The blue line represents the rarefaction curve for the human gut microbiome, while the red line corresponds to the rarefaction curve for the dog gut microbiome. Each curve illustrates the observed number of ASVs at varying sequencing sample sizes. ASV, amplicon sequence variant


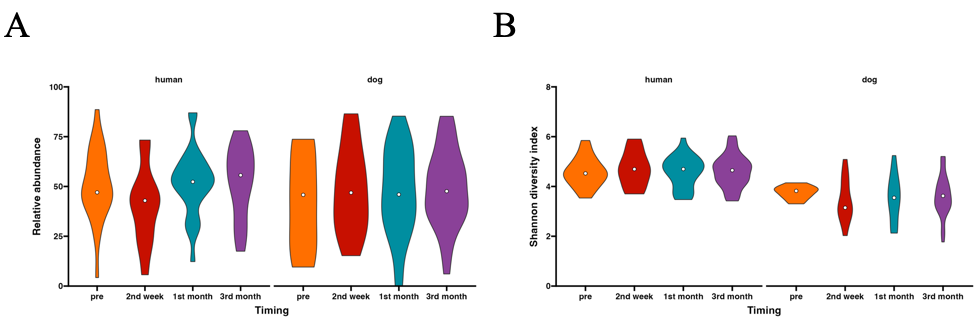


Supplementary Figure 2. The composition and diversity of intestinal microbes in humans and dogs. The composition and diversity of intestinal microbes in humans and dogs before and after cohabitation. (A) Violin plots of total relative abundance of the top five dominant genera in each host at pretest (human: n = 24, dog: n = 10), 2^nd^ week (n = 12), 1^st^ month (n = 25), and 3^rd^ month (n = 28). (B) The Shannon diversity index in each host at each specified time point is indicated.

1. Supplementary Tables

Supplementary Table 1. Statistically significant ANCOM results at the genus level. Relative abundance across all samples or ASVs within a group was summed.

|  |  |  |  | Number of features (100 percentile) | Number of features (100 percentile) |
| --- | --- | --- | --- | --- | --- |
| ASV ID | Taxonomy | clr | W-statistic value | human | dog |
| 85ed0d0f579237bd0a2f8b98c28b3a5c | *Peptoclostridium* | 2.88 | 366 | 1 | 3803 |
| 27e2aa13ac8d9a01ddf2863741c5cbcb | *Blautia* | 2.672 | 360 | 1 | 5677 |
| fa18371d7062793ea19a315790b69117 | *Peptoclostridium* | 2.495 | 360 | 641 | 5657 |
| 8e7b50632385bb16ea32e2ae7f5c1a50 | *Fusicatenibacter* | -1.9501 | 351 | 2067 | 1 |
| 1c911e95f53361d9972bbd73b40ea416 | *Blautia* | 1.599 | 331 | 449 | 1670 |
| fb7ae9e4b7fb258deaba2e96fe851d32 | *Lachnospiraceae sp.* | 1.675 | 331 | 1 | 1725 |

**ANCOM, analysis of composition of microbiomes; ASV, amplicon sequence variant**

**Supplementary Table 2. Relative abundance of shared ASVs at the sampling time for pairs sharing ASVs.**

| ASV ID | pair | timing | abundance of human sample | abundance of dog sample |
| --- | --- | --- | --- | --- |
| ASV_001 | Pair_A | pre | 0 | 0 |
| ASV_001 | Pair_A | 1st month | 0 | 3.782 |
| ASV_001 | Pair_A | 3rd month | 11.008 | 2.422 |
| ASV_001 | Pair_B | pre | 0 | 0 |
| ASV_001 | Pair_B | 1st month | 1.072 | 2.554 |
| ASV_001 | Pair_B | 3rd month | 0 | 0 |
| ASV_002 | Pair_C | pre | 0 | 0 |
| ASV_002 | Pair_C | 1st month | 12.661 | 10.383 |
| ASV_002 | Pair_C | 3rd month | 0 | 6.059 |
| ASV_002 | Pair_D | 2nd week | 0 | 0 |
| ASV_002 | Pair_D | 1st month | 1.844 | 20.006 |
| ASV_002 | Pair_D | 3rd month | 0 | 13.442 |
| ASV_003 | Pair_E | 2nd week | 5.091 | 0.0 |
| ASV_003 | Pair_E | 1st month | 2.703 | 2.349 |
| ASV_003 | Pair_E | 3rd month | 0.0 | 0.0 |
| ASV_004 | Pair_E | 2nd week | 0.0 | 0.0 |
| ASV_004 | Pair_E | 1st month | 1.976 | 7.468 |
| ASV_004 | Pair_E | 3rd month | 0 | 0 |
| ASV_005 | Pair_C | pre | 0 | 0 |
| ASV_005 | Pair_C | 1st month | 4.470 | 16.124 |
| ASV_005 | Pair_C | 3rd month | 1.646 | 0 |
| ASV_006 | Pair_D | 2nd week | 0 | 0 |
| ASV_006 | Pair_D | 1st month | 1.293 | 0 |
| ASV_006 | Pair_D | 3rd month | 1.705 | 1.745 |
| ASV_007 | Pair_F | pre | 0 | no sample |
| ASV_007 | Pair_F | 1st month | 9.025 | 8.733 |
| ASV_007 | Pair_F | 3rd month | 0 | 0 |
| ASV_008 | Pair_G | 2nd week | 6.487 | 0 |
| ASV_008 | Pair_G | 1st month | 0 | 0 |
| ASV_008 | Pair_G | 3rd month | 5.081 | 5.130 |
| ASV_009 | Pair_C | pre | 0 | 0 |
| ASV_009 | Pair_C | 1st month | 0 | 4.624 |
| ASV_009 | Pair_C | 3rd month | 2.150 | 2.504 |
| ASV_010 | Pari_H | pre | 0 | 0 |
| ASV_010 | Pari_H | 1st month | 0 | 0 |
| ASV_010 | Pari_H | 3rd month | 13.069 | 56.772 |
| ASV_011 | Pair_I | 2nd week | 0 | 0 |
| ASV_011 | Pair_I | 1st month | 0 | 0 |
| ASV_011 | Pair_I | 3rd month | 1.545 | 1.157 |

**ASV, amplicon sequence variant**

**Supplementary Table 3.** The ratio of samples in which ASVs with a detection rate of > 1% were confirmed in humans and dogs.

| ASV ID | human samples | dog samples |
| --- | --- | --- |
| ASV_001 | 0.079 | 0.31 |
| ASV_002 | 0.079 | 0.29 |
| ASV_003 | 0.045 | 0.013 |
| ASV_004 | 0.045 | 0.013 |
| ASV_005 | 0.023 | 0.387 |
| ASV_006 | 0.023 | 0.027 |
| ASV_007 | 0.045 | 0.027 |
| ASV_008 | 0.023 | 0.053 |
| ASV_009 | 0.023 | 0.187 |
| ASV_010 | 0.034 | 0.147 |
| ASV_011 | 0.011 | 0.013 |
